# Quantitative visualization of lignocellulose components in transverse sections of moso bamboo based on FTIR macro- and micro-spectroscopy coupled with chemometrics

**DOI:** 10.1186/s13068-018-1251-4

**Published:** 2018-09-26

**Authors:** Xiaoli Li, Yuzhen Wei, Jie Xu, Ning Xu, Yong He

**Affiliations:** 1 College of Biosystems Engineering and Food Science, Zhejiang University, 866 Yuhangtang Road, Hangzhou, 310058 China; 2 College of Biological Chemical Science and Engineering, Jiaxing University, 1 Jiahang Road, Jiaxing, 314001 China; 3 Institute of Drug Development and Chemical Biology, Zhejiang University of Technology, 18 Chaowang Road, Hangzhou, 310014 China

**Keywords:** Moso bamboo, FTIR microscopic imaging, Lignocelluloses, Calibration transfer, Multivariate quantitative calibration

## Abstract

**Background:**

Due to the increasing demands of energy and depletion of fossil fuel, bamboo is considered to be one of the most important renewable biological resources on the basis of its advantages of rapid growth ability and rich reserves. Cellulose, hemicellulose, and lignin are the three most important constituents in moso bamboo. Their concentrations and, especially, their microscopic distributions greatly affect their utilization efficiency and other physical properties as a biomass resource. However, no studies have achieved a quantitative visualization of the distribution of lignocellulose concentrations in transverse sections of bamboo. Therefore, this study proposed the use of quantitative multivariate spectral analysis to reveal the micro-chemical distribution of lignocelluloses in bamboo based on an integration of FTIR macro- and micro-spectroscopic imaging techniques.

**Results:**

Multivariate calibration models for the quantitative determination of lignocelluloses of bamboo were developed based on FTIR macro-spectroscopy, and the quantitative calibration models based on the FTIR characteristic bands showed an excellent performance with determination coefficients of 0.933, 0.878, and 0.912 for cellulose, hemicellulose, and lignin, respectively. These quantitative models were then utilized to the FTIR micro-spectroscopy of bamboo transverse sections which were corrected using a direct standardization algorithm. Subsequently, the micro-chemical distributions of cellulose, hemicellulose, and lignin were obtained based on the integration of the multivariate calibration models and corrected FTIR micro-spectroscopy. The combination of the multivariate calibration models and calibration transfer algorithm resulted in a final quantitative visualization of the chemical distributions of lignocelluloses in moso bamboos.

**Conclusions:**

Integration of the FTIR macro- and micro-spectroscopic imaging techniques can provide comprehensive information that can be used to exploit the resource of moso bamboo to develop biofuels and biosynthetic materials.

## Background

With the merits of rapid growth, high productivity, rich germplasm resources, and low ash content and alkali index, bamboo has been considered to be one of the most important renewable biological resources [1]. Moso bamboo is the most widely cultivated bamboo in China, with approximately 65% of the total area of bamboo forest [2]. The carbon absorbed by moso bamboo is mainly stored in three important types of biomass, namely, cellulose, hemicellulose, and lignin, which make up over 90% of the total dry mass [3]. Understanding the unique multilayer structure and fine-compositional distribution of these components of bamboo would provide very important information on its function. As sustainable materials in biomass energy, lignocelluloses, and their microscopic crosslinking structure have very important effects on the biomass utilization with respect to processes such as chemical and enzymatic pretreatment, hydrolysis, and fermentation [4]. Imaging of lignocellulose distribution within the micro-multilayer structure is, therefore, not only helpful for understanding the growth mechanism of bamboo from the perspective of accumulation of the biomass, but also has significance as an important guide in the study of biological energy transformation [5]. There is enormous diversity in the lignocellulosic components and their microscopic structure among bamboo samples obtained from different locations or physiological ages [6], as well as among the various physiological parts within an individual bamboo culm. Hence, the real-time monitoring of lignocelluloses is of great importance for the optimization of biomass treatment [7]. Nevertheless, no research regarding the quantitative visualization of lignocellulosic content of bamboo at microscopic view has been reported so far.

The FTIR micro-spectroscopic imaging technique is an excellent monitoring method that has been used to examine the lignocellulose micro-distribution in the crop stalk [8], the chemical distribution of the composition of the adaxial surface of Ginkgo biloba leaves [9], the chemical composition of calcified deposits of prostatic calculi and calcific tendonitis [10], molecular structure of wood [11], the carbohydrate excipients in granules of traditional Chinese medicines [12], and the effects of three heat treatments on cotyledon tissues [13].

However, most of these studies examined the chemical compositional distribution by mapping integrated areas or the intensity of a diagnostic spectral band of the compound. This strategy is based on two assumptions: (1) the concentration of the compound is the only factor that determines the intensity of the well-defined spectral band associated with the compound [14] and (2) there is a significant and completely linear relationship between the concentration of compound and the spectral intensity at diagnostic band. However, these two assumptions are often invalid when the well-defined band overlaps with those of other compounds or when the bands broaden or shift in response to a chemical change. Moreover, these assumptions mean that the strategy is sensitive to interferences induced by discrepancies of spectral collection condition, sample preparation, and spectral pretreatment. In other words, because only the intensity of a diagnostic band is monitored, broadening and shifts are easily interpreted as intensity changes.

Therefore, more reliable results could be achieved utilizing a set of bands, i.e., the concentration of a complicated compound would be determined by a synergistic combination of multiple diagnostic bands. However, it is challenging to describe the quantitative relationship between a set of FTIR micro-spectral bands and the concentration of compounds. Because FTIR micro-spectroscopy involves micro-spatially resolved spectral response profiles and the concentration of compound at each pixel cannot realistically be measured using traditional analytical chemistry [15], there is not a practical method to establish quantitative relationship between FTIR micro-spectroscopy and concentration of a compound at each pixel at the micro-level. However, it is worth noting that a quantitative relationship between FTIR macro-spectroscopy and the concentration of the compounds can be developed, because the concentration of compound at macro-level can be obtained through homogenization of the tissue [16]. Therefore, the quantitative relationship between the FTIR spectroscopic characteristics and compounds of interest may be expanded by transferring the calibration from a master instrument (FTIR macro-spectroscopy) to a slave instrument (FTIR micro-spectroscopy).

Calibration transfer is one of the most commonly applied means of compensating for spectral variations, which may be caused by many factors such as the surface texture, granularity, and change or aging of instruments [17]. The multivariate transfer calibration technology of direct standardization (DS) has been successfully applied to the quantitative analysis of creatinine in serum samples by transferring the calibrations of two image analysis instruments [18] and spectral matching and combining of nuclear magnetic resonance spectral data set from different instruments [19, 20]. Multivariate calibration transfer has also been used to transform NIR spectral and instrumental artifacts to reduce the prediction error [21] and to correct the NIR spectral measurement variation between one dispersive instrument and one Fourier transform (FT) instrument for quantitative analysis of fish meal mixed with soybean meal [22]. Calibration transfer from the reflectance mode data from a conventional Fourier transform infrared (FTIR) spectrometer to a portable FTIR spectrometer in transmission mode has been realized by adopting the simple strategy of DS for the prediction of quality parameters of diesel/biodiesel blends [23]. The above studies have proven the feasibility of calibration transfer to eliminate the spectral measurement variability among different spectral instruments.

Based on the above discussion, we created a novel scheme to achieve the quantitative visualization of the lignocellulosic content of bamboo at the microscopic level. Specifically, we first established the determination models for lignocellulosic components based on FTIR macro-spectroscopy, and then, a DS algorithm was performed to correct the spectral variation between FTIR micro-spectroscopy and FTIR macro-spectroscopy. After that, the determination models based on macro-spectroscopy were applied to the corrected FTIR micro-spectroscopy. Consequently, the quantitative visualization of the lignocellulosic content of transverse sections was finally achieved. To the best of our knowledge, this scheme for quantitative visualization at microscopic view has not yet appeared in the previous literature.

## Methods

### Sample preparation

The moso bamboo samples were collected from three sites: Maoyang (MY) Village (E: 119.394, N: 27.727), Jingning County, Zhejiang Province; Baitanao (BT) Village (E: 119.330, N: 27.827), Jingning County, Zhejiang Province; Daishi (DSH) village (E: 106.670, N: 30.415), Guangan County, Sichuan Province. For each site, three culms of bamboo with each physiological age were collected: accordingly, a total of 15 culms of five physiological ages (1–5 years) were obtained. In addition, four positions from each culm including base, middle, top, and middle node sections were sampled. Thus, 3 (sites) × 5 (ages) × 4 (parts) × 3 (replicates) = 180 samples were obtained for analysis.

The samples were prepared using various treatment methods for the macro-lignocellulosic content measurement and microstructure chemical imaging of lignocelluloses of bamboo. The specific treatment procedures are described below.

For macro-lignocellulosic content analysis, all of the collected moso bamboo samples were first air-dried. Then, a series of sections were cut from the culms of bamboo, and these sections were subsequently cut into small pieces. Next, these pieces were ground with a grinder (Tissuelyser-48, Shanghai Jingxin Industrial Development Corporation, China) to obtain the moso bamboo powder. To ensure the accuracy of chemical measurement and spectra collection [24–26], the powder was sequentially sifted through a 380 μm mesh screen and a 250 μm mesh screen. Finally, the powder with particle size between 250 and 380 μm was collected for FTIR spectral acquisition and reference chemical measurement.

For the microstructure chemical imaging analysis, the middle internodes of the moso bamboo samples from the three sites at the second physiological age were selected. The middle internodes of the culms were cut into strips, and then, the strips were sliced into 15 μm transverse sections using a rotary microtome (KD-1508A, Zhejiang Jinhua Kedi Instrumental Equipment Corporation, China). Transverse sections without fragmentation and curling were selected.

### FTIR macro- and micro-spectroscopy acquisition

For the FTIR macro-spectroscopic analysis, the bamboo powder was mixed with a KBr pellet (spectral purity, SP, Sinopharm Chemical Reagent Corporation, China) at a ratio of 1 to 49 and then ground sufficiently to allow the formation of tablets using a pressure machine (FY-15, Tianhe Machinery Equipment Corporation, Shanghai, China) with 15 MPa pressure and 30 s duration. The FTIR spectra of these tablets were then collected using an FTIR spectrometer (FTIR 4100, JASCO Corporation, Japan) in transmittance mode with a spectral range of 350–7800 cm^−1^ and spectral resolution of 4 cm^−1^. During the spectral collection, the repetition scan times for each sample were set to 32, and the background signal sampling interval was set to 45 min.

**Fig. 1:**
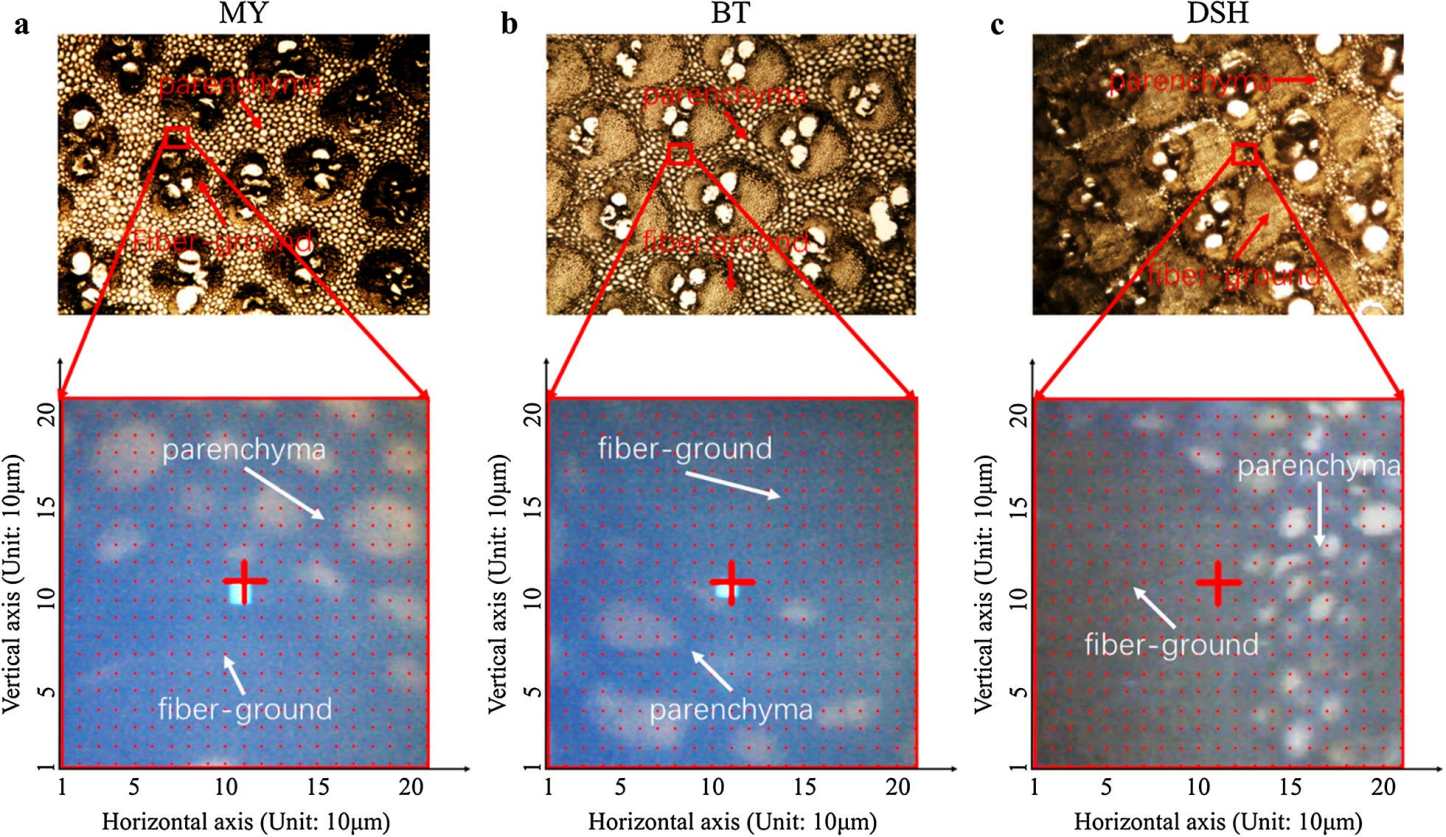
Scanning area maps of transverse sections from various product sites, with a microscopy in ×40 objective lens. MY, BT, and DSH indicate the specific growth sites Maoyang, Baitanao, and Daishi (similarly hereinafter)

For the FTIR micro-spectroscopy collection, the prepared transverse sections were first examined using a microscope (CX31, Olympus Corporation, Japan) to evaluate the quality of slices. The qualified slices that demonstrated better tissue integrity with vascular bundle and parenchyma features [8] were then selected and scanned using an FTIR microscopic imager (Nicolet iN10, Thermo Fisher Scientific, US), as shown in Fig. 1. When scanning the transverse section, a liquid nitrogen-cooled mercury cadmium telluride detector was chosen to capture the FTIR micro-spectroscopy with the range of 675–4000 cm^−1^ in transmittance mode. Based on the information richness, scanning speed, and instrument performance, the spectral and spatial resolution were set to 8 cm^−1^ and 10 μm × 10 μm, respectively. To maximize the signal-to-noise ratio, the repetition scanning times were set to 256. To reduce the interference caused by background changing, a background spectrum was collected every 45 min. For each transverse section, the central area of the ground tissue including fiber strands, parenchymal cell, and their boundary regions with area of 210 μm × 210 μm was scanned. Accordingly, 441 spectra with 21 × 21 pixels were obtained.

### Chemical measurement

The concentrations of hemicellulose, cellulose, and lignin were measured according to Van Soest method [27]. Before measurement, 0.5 g powder was accurately weighed for each sample [28]. Then, the quantified powder was subjected to four main steps: neutral detergent washing, acid detergent washing, 72% sulfuric acid washing, and ashing in a muffle furnace. At each step, the samples were weighed at least three times to ensure the relative error is lower than 5%. All reagents used for the chemical measurement were at least grade of analytical reagent (AR). Specifically, the main reagents used in this experiment were as follows: sodium dodecyl sulfate (AR), anhydrous sodium dihydrogen phosphate (AR), concentrated sulfuric acid (AR), cetyltrimethyl-ammonium bromide (AR), and acetone (AR) were obtained from Sinopharm Chemical Reagent Corporation (Shanghai, China), and decalin (AR), sodium sulfite (guaranteed reagent, GR), and edathamil disodium (GR) were obtained from Alfa Aesar Corporation (Ward Hill, MA, US). To ensure the accuracy of the experimental results as far as possible, a water purification system (EPED-E2-10TJ, Yipuyida Science and Technology Development Corporation, Nanjing, China) was used to produce the ultra-pure water for solution preparation.

### Chemometrics

#### Multivariate calibration analysis

##### Sample allocation and abnormal sample elimination

Partial least-squares regression (PLSR), one of the most widely used multivariate calibration methods [29], was used to establish the quantitative determination model of lignocelluloses. Before modeling, all samples were divided into a calibration set and a prediction set. Specifically, all of the samples were first sorted in ascending order on the basis of their chemical concentration. Second, each three consecutive samples were treated as a subset. From each subset, the median sample was taken to form prediction set, and the remaining samples were used as calibration samples.

Sample and measurement abnormalities always have a serious negative impact on building models. Therefore, it is necessary to detect and remove the abnormal samples. Monte Carlo sampling (MCS) method [30] was adopted to detect abnormal samples in this study. This method is based on the sensitivity of the prediction error to abnormal samples. The spectral and the concentration information are used for this purpose, and the specific algorithm was as follows: first, the best number of latent variables for the PLSR was calculated; second, the proportion of calibration set to validation set and the cycle index for sampling were set; third, the PLSR model was established to predict the validation set, in which the prediction error distribution of each sample is predicted through multiple cycles; and fourth, the mean and standard deviation (STD) of predicted residual for each sample were determined. The abnormal samples can be detected by inspection of the mean and STD distribution diagram.

##### Quantitative determination of lignocelluloses with characteristic bands

The information provided by FTIR spectroscopy is rich. However, the information often includes redundancy. The information redundancy reduces the model stability and increases the computational complexity. To reduce the information redundancy, two strategies were utilized in succession. The first strategy was to select spectral regions using the interval partial least-squares (iPLS) method. The second strategy was to select spectral bands from the characteristic range using the competitive adaptive reweighted sampling (CARS) algorithm.

The iPLS aims to extract important spectral regions and eliminate interferences from other regions by establishing a set of local PLSR models on equidistant subintervals of the full-spectrum region [31]. Thereafter, a model with the lowest root mean square error of cross validation (RMSEV) is selected as the optimal spectral region. Furthermore, different combinations of regions are also used to develop PLSR model, and the optimal combination is the one with the lowest RMSEV [32].

The selection of spectral bands based on the CARS algorithm [33] includes four main steps: first, samples were randomly selected based on the MCS method to constitute the calibration and validation sets according to a certain proportion, and a PLSR model was built subsequently for each loop; second, bands with relatively small absolute values of the regression coefficients as indicated by the exponential decay function for each cycle were removed; third, the bands were further screened by evaluating their weights; and fourth, the RMSEVs of the band subsets generated from each cycle were compared, and the band subset with the lowest RMSEV is the final result.

#### Spectra transfer

##### Spectral matching and interpolation

For model transfer, the spectra acquired by the Jasco spectrometer (treated as the master instrument) were trimmed to 675–4000 cm^−1^ to match the spectral range of the Nicolet micro-spectrometer (treated as the slave instrument). Then, a cubic spline interpolation was performed to define the fitting values for the spectra captured by the slave instrument to form the same spectral interval as the master instrument. Thus, through trimming and interpolation, 2800 spectral variables per spectrum were obtained for transfer in the range of 675–4000 cm^−1^ for both master and slave instruments [34].

##### Spectral transfer by direct standardization

The direct standardization (DS) algorithm is a well-accepted means to eliminate spectral variation and compute the transfer parameters between master and slave instruments by estimating the difference between the detection processes. This involves a straightforward strategy to perform the transfer of the parameters from the slave instrument to the master instrument. According to the research of Bouveresse and Massart [35], it is important to choose representative samples to define the differences between the master and slave instruments. In this study, the samples from the middle part of the 2 year physiological age subset from the three sites were collected, using three samples for each site. For the spectra acquired by master instrument, the mean spectrum of the samples from a site was taken as a representative spectrum, so three representative spectra were prepared from the master instrument. For the slave instrument, the micro-spectroscopic hypercubes of the transverse slices corresponding to the three sites were averaged to generate three representative spectra. The representative spectra of master and slave instruments was adopted to compute the DS transfer model.

The parameters for DS transfer model can be expressed through the following formula (1):

1 $$X_{\text{master}} = X_{\text{slave}} *E + B.$$

In the formula, *X*_master_ means the spectral matrix of the standardization samples from the master instrument, which corresponds to the macro-average spectra acquired by Jasco FTIR spectrometer. *X*_slave_ denotes the spectral matrix from the slave instrument, which specifically refers to the micro average spectra obtained by Nicolet FTIR micro-spectrometer aforementioned. *E* simply indicates the transfer matrix from slave instrument to master instrument. *B* represents the residual for model compensation.

#### Infrared micro-spectroscopic imaging of lignocelluloses

After a quantitative relationship between the FTIR spectroscopy and lignocelluloses of bamboo was defined based on the multivariate calibration analysis, the quantitative determination model was imported to the corrected FTIR micro-spectroscopy using the direct standardization (DS) method. Subsequently, the concentration of lignocelluloses at each pixel can be generated based on this integration of the multivariate calibration model and calibration transfer. Finally, lignocellulose distribution maps of the bamboo transverse sections can be obtained.

## Results and discussion

### Overview of the lignocelluloses of moso bamboos

**Fig. 2:**
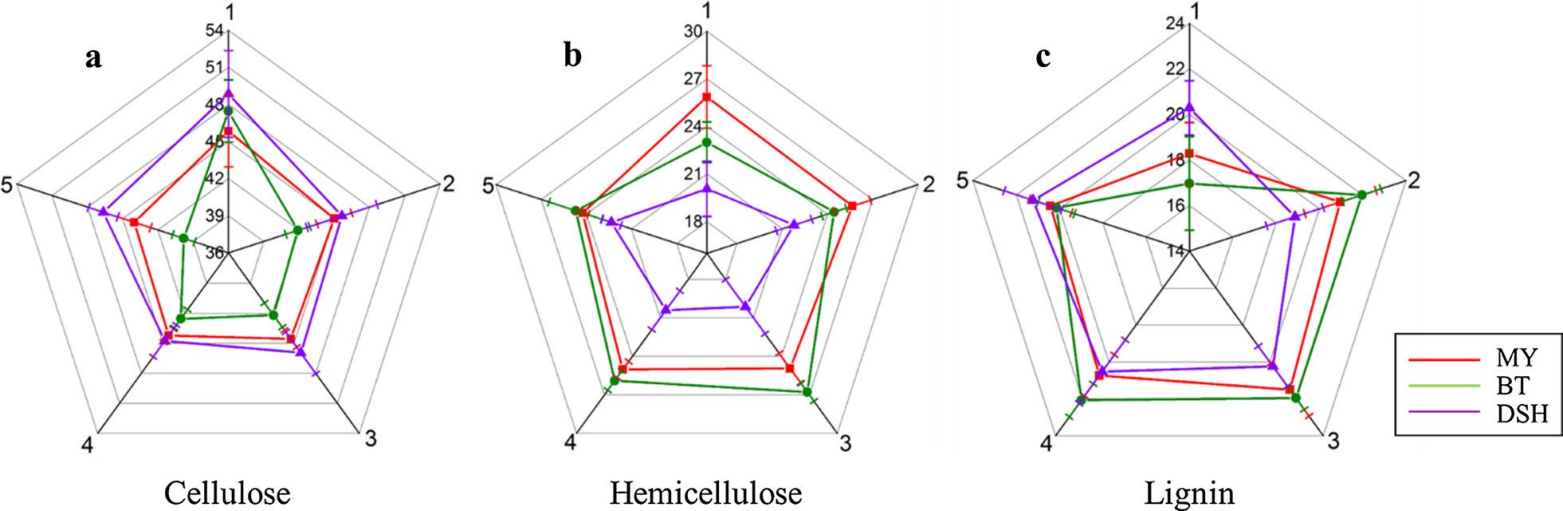
Cobweb maps for the cellulose, hemicellulose, and lignin concentrations in different product sites and ages. In this figure, the numbers ‘1’ to ‘5’ arrayed around the cobweb map refer to the age; the growth sites were denoted by the lines with different colors, and the numbers along the vertical radius represent the percentages of lignocellulosic content

To explore the distribution characteristics of the lignocelluloses of samples from the various sites, ages, and parts, the lignocellulose concentration values for the middle parts of samples from three sites and five ages are shown in Fig. 2. Furthermore, all 180 moso bamboo samples were analyzed by a multivariate analysis of variance.

For cellulose, the *p* values for the sites, years, and parts were 0.000, 0.000, and 0.000, respectively, and all of which were less than 0.05. These values indicated that there were significant differences in the cellulose content for the various sites, years, and parts. In addition, Duncan’s multiple range test (MRT) was used to further study the differences in the cellulose content among the different sites. It was found that the contents of cellulose in MY, BT, and DSH were significantly different from each other, and the cellulose content from high to low was in the order of DSH, BT, and MY. Duncan’s MRT was also used to analyze the influence of growing years on cellulose content. The content of cellulose in 1-year-old moso bamboo was significantly higher than that in the 2nd–5th years, whereas the content of cellulose was not significantly different among 2nd, 3rd, 4th, and 5th years. To analyze the differences in the cellulose content among the base, middle, top, and node, Duncan’s MRT was used to analyze the influence of part on cellulose content. It was found that the nodes had a significantly lower cellulose content than the bases, middles, and tops. Furthermore, the cellulose content of top was significantly different from those in the base, middle, and node and was higher than that of node. A graphical display of the cellulose contents among different sites and years is shown in Fig. 2a.

For hemicellulose, the *p* values for the sites, years, and parts were 0.000, 0.000, and 0.028, respectively, and all of which were less than 0.05. These values indicated that there were significant differences in the hemicellulose content for the different sites, years, and parts. In addition, Duncan’s new MRT was used to further study the differences in the hemicellulose content among the various production areas. It was found that the content of hemicellulose for the DSH samples was significantly different from those of MY and BT, among which the content of hemicellulose in DSH was the lowest, whereas the difference between MY and BT was not significant. Duncan’s MRT was also used to analyze the influence of growing years on hemicellulose content. The content of hemicellulose in 1-year-old moso bamboo was significantly lower than that in 2nd–5th years, whereas the content of hemicellulose was not significantly different among 2nd, 3rd, 4th, and 5th years. To analyze the difference of hemicellulose content among the bases, middles, tops, and nodes, Duncan’s MRT was used to analyze the influence of position on hemicellulose content. It was found that the hemicellulose content of the nodes was significantly higher than those in the bases, middles, and tops. A graphical display of the hemicellulose contents among different sites and years is shown in Fig. 2b.

In terms of lignin, the *p* values for sites, years, and parts were 0.349, 0.000, and 0.000, respectively. These values indicated that there were significant differences in the lignin content in different years and parts, whereas the lignin content in different sites was not significantly different. Duncan’s MRT was also used to analyze the influence of growing years on lignin content. The content of cellulose in 1-year-old moso bamboo was significantly lower than that in 2nd–5th years, whereas the content of lignin was not significantly different among 2nd, 3rd, 4th, and 5th years. To analyze the differences in the lignin content among the bases, middles, tops, and nodes, Duncan’s MRT was used to analyze the influence of part on lignin content. It was found that the lignin content of the nodes was significantly higher than those in the bases, middles, and tops. An graphical display of the lignin contents among different sites and years is shown in Fig. 2c.

Statistical analysis of lignocellulosic components verified the existence of differences in growth areas, ages, and parts. It is, therefore, necessary to consider these factors when utilizing bamboo as biomass resources. Furthermore, the statistical analysis also highlights the importance of quantitative visualization for lignocellulosic components, because it provides an ideal way to illustrate the differences in a visual manner.

### Analysis of FTIR spectroscopy of bamboo in macroscale

**Fig. 3:**
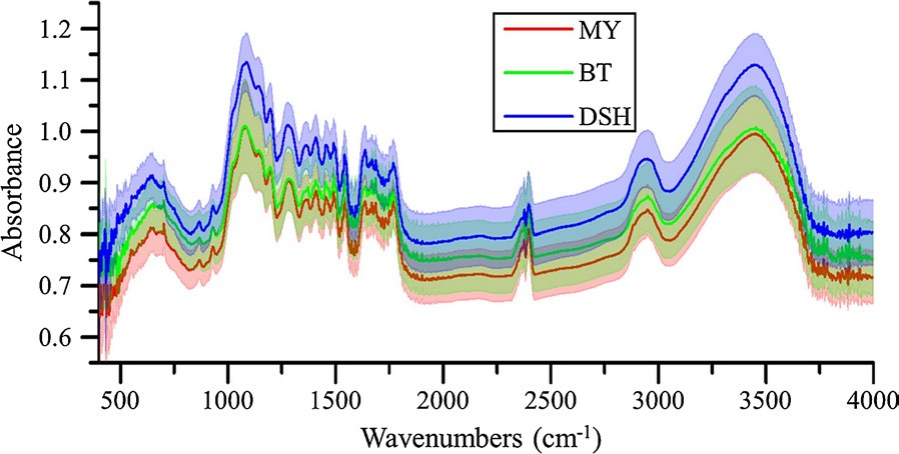
Average spectra ± STD of the samples from the different sites. The line with dark color represents the average spectra. The upper and lower boundaries of the translucent areas represent ± STD

The average spectra ± STD of the samples from different sites are shown in Fig. 3. These results show that noise can be observed easily in the front and rear regions of IR spectroscopy, so the wavelength range of 881–3581 cm^−1^ was selected as the effective range for further analysis. Figure 3 also shows that the spectral profiles of the various samples were basically consistent. A gradient trend in the absorbance was observed, which indicated that the internal composition of these samples was almost identical, but there were differences in the specific content. This fact provided the premise for establishment of spectral quantitative determination model of the internal composition.

With respect to a more detailed analysis of the spectroscopy, the absorption peak at 3449 cm^−1^ (between 3200 and 3650 cm^−1^) mainly reflects the –OH-stretching vibration [36]. The absorption peak at 2946 cm^−1^ (between 2500 and 3200 cm^−1^) is mainly caused by the stretching vibration of C–H [37]. For the 2000–2500 cm^−1^ range, the peaks at 2397, 2377, and 2363 cm^−1^ are the result of CO_2_ that was not fully deducted from the background of the air [38]. Regarding the 1500–2000 cm^−1^ range, the peaks at 1592, 1639, and 1735 cm^−1^ correspond to in-plane C=C aromatic vibration, O–H bending of absorbed water, and C=O stretching in ester groups, respectively [39]. This region is the most important carbonyl absorption region. In the interval of 1300–1500 cm^−1^, the peaks at 1364 and 1455 cm^−1^ mainly provide information on the C–H-bending vibrations [40, 41]. All of the single bond-stretching vibration frequencies and vibration frequencies of the molecular skeleton are in the 910–1300 cm^−1^ region [42].

### Quantitative determination of lignocelluloses based on multivariate calibration analysis

#### Sample division and preliminary modeling analysis

Through the allocation method mentioned in “Sample allocation and abnormal sample elimination”, a calibration set with 120 samples and a prediction set with 60 samples were obtained. The statistical results of the concentration values are shown in Table 1.

**Table 1:** Statistical results for lignocellulose concentrations

Constituent	Set	Number	Maximum	Minimum	Average	STD
Hemicellulose	Calibration	120	0.282	0.177	0.237	0.025
	Prediction	60	0.282	0.179	0.237	0.025
	Total	180	0.282	0.177	0.237	0.025
Cellulose	Calibration	120	0.538	0.380	0.446	0.029
	Prediction	60	0.537	0.381	0.446	0.029
	Total	180	0.538	0.380	0.446	0.029
Lignin	Calibration	120	0.239	0.138	0.204	0.019
	Prediction	60	0.236	0.145	0.204	0.019
	Total	180	0.239	0.138	0.204	0.019

The numbers in the columns of maximum, minimum, average, and STD represent the percentages of lignocellulosic content

Table 1 shows that the concentration ranges of the calibration set covered those of the prediction set. The average values and STD of the prediction and calibration sets were very close for hemicellulose, cellulose, and lignin, which indicated that the allocation method was suitable.

After sample allocation, three PLSR models were built with the full-range (881–3581 cm^−1^) spectroscopy for hemicellulose, cellulose, and lignin, and the performances of these models are shown in Table 2.

**Table 2:** Performances of PLSR regression models based on full bands

Constituent	LVs	*R*_C_^2^	RMSEC	*R*_P_^2^	RMSEP	RPD
Hemicellulose	16	0.850	0.010	0.780	0.012	2.131
Cellulose	17	0.918	0.008	0.836	0.012	2.466
Lignin	17	0.906	0.006	0.802	0.008	2.248

LVs means the number of latent variables, *R*_C_^2^ and *R*_P_^2^ indicate the determination coefficients for the calibration and prediction, respectively, RMSEC and RMSEP represent the root mean square errors for the calibration and prediction, respectively, and RPD denotes the ratio of performance to deviation (similarly hereinafter)

The results in Table 2 show that there was a strong correlation between the IR spectroscopy and the concentration of lignocellulose, but the performance was not fully optimized, so further analysis was needed to determine whether there were any other interferences.

#### Elimination of abnormal samples

**Fig. 4:**
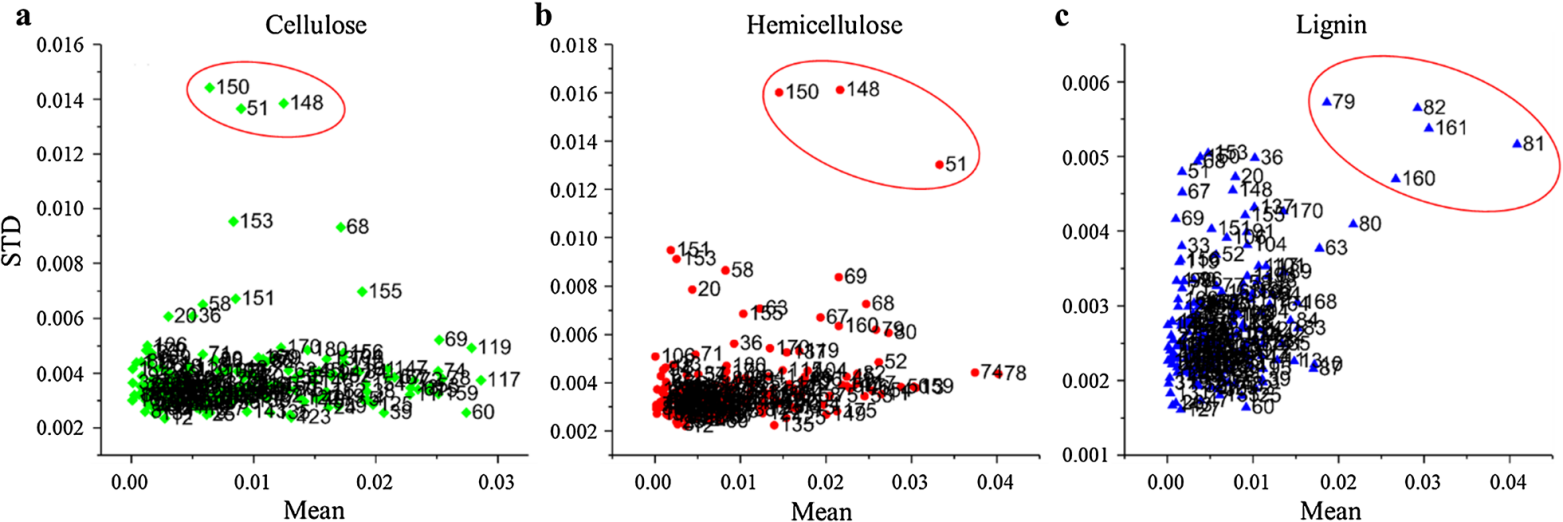
Prediction residual mean values versus STD for all samples through MCS method. **a** Cellulose, **b** hemicellulose, and **c** lignin. In each subgraph, the abscissa and the ordinate represent the mean and STD of prediction residual, respectively. The number on the right of each point denotes the serial number of each sample

Because many factors such as unstable instrument status and imperfect operation would produce abnormal samples, the MCS method was performed to detect abnormal samples. When executing the MCS method, the number of cyclic sampling was set to 5000 times, and the proportion between calibration and validation was set to 4:1. After execution, the scatter plot of the prediction residual mean values versus STD is shown in Fig. 4.

The outlier points can be considered to be abnormal samples, because they were not stable or applicable for the models built based on the rest of the samples. According to the MCS evaluation, samples 51, 148, and 150 were removed for the modeling of hemicellulose and cellulose, and samples 79, 81, 82, 160, and 161 were removed for lignin. After elimination of the outlier samples, PLSR models were built based on the rest of the samples for hemicellulose, cellulose, and lignin. The results are shown in Table 3. Compared with Table 2, the performances of the models were improved for all the three lignocelluloses, especially for lignin. This phenomenon indicated that the MCS method effectively detected the abnormal samples.

**Table 3:** Performances of PLSR regression models after elimination of abnormal samples

Constituent	LVs	*R*_C_^2^	RMSEC	*R*_P_^2^	RMSEP	RPD
Hemicellulose	17	0.896	0.008	0.789	0.012	2.178
Cellulose	18	0.936	0.007	0.852	0.011	2.599
Lignin	16	0.932	0.004	0.913	0.005	3.392

#### Selection of characteristic FTIR spectral bands

**Fig. 5:**
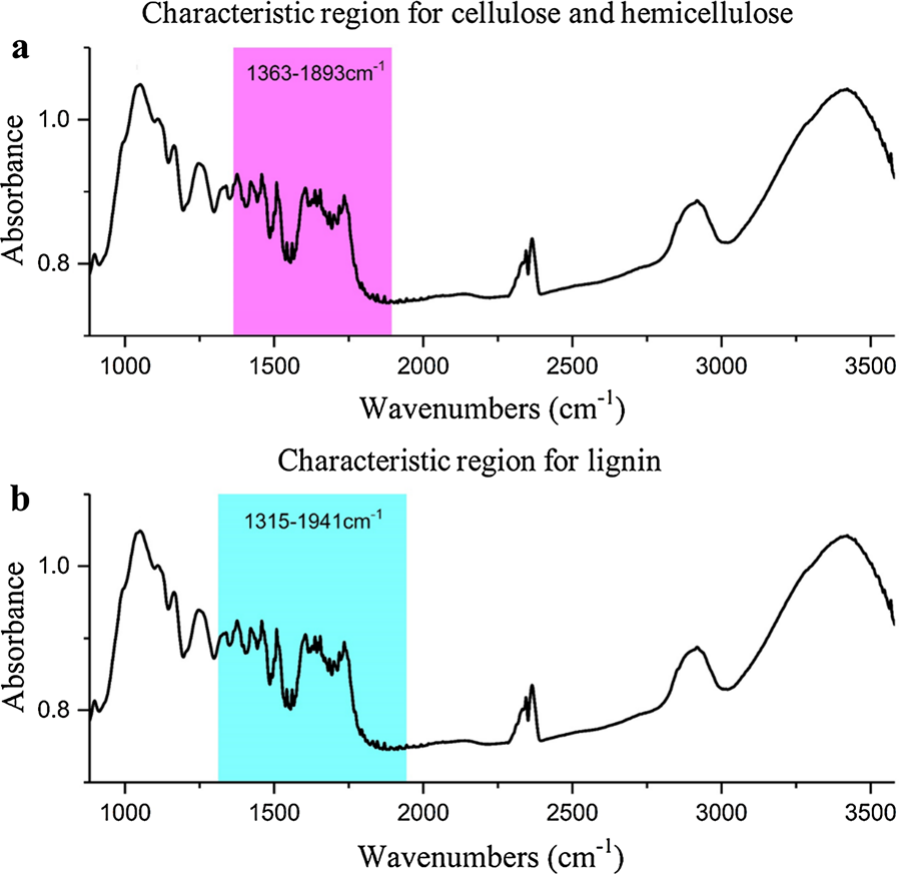
Important spectral sections for lignocellulose modeling based on iPLS. **a** Hemicellulose and cellulose, **b** lignin

FTIR spectroscopy produces rich information, but this also results in a problem of redundancy. To reduce the interference of the unrelated intervals for modeling, an iPLS method with a window width of 99 was performed to select the important spectral regions. The corresponding selected sections for the three lignocelluloses are shown in Fig. 5. In addition, the PLSR models based on selected regions were subsequently established, and the results of these models are shown in Table 4.

**Table 4:** Performance of PLSR models based on characteristic spectral ranges

Component	Range	LVs	*R*_C_^2^	RMSEC	*R*_P_^2^	RMSEP	RPD
Hemicellulose	1363–1893	16	0.863	0.009	0.814	0.011	2.316
Cellulose	1363–1893	15	0.892	0.010	0.864	0.011	2.709
Lignin	1315–1941	12	0.934	0.004	0.915	0.005	3.422

Table 4 shows that the performances of the PLSR models have been improved compared with those in Table 3 with approximately 75% reduction in the dimensions of independent variables (spectral bands). Although the improvement was small, this reduction of dimension greatly reduced the complexity of the models.

**Fig. 6:**
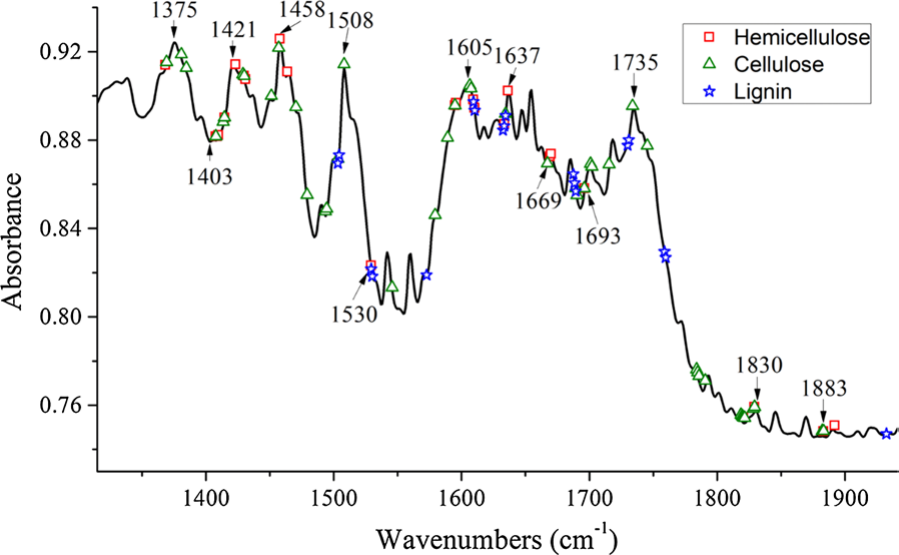
Characteristic spectral bands of lignocelluloses. For the different components, the characteristic bands were marked with different symbols. The square, triangle, and pentagon correspond to hemicellulose, cellulose, and lignin, respectively

It is worth noting that the selected ranges for lignocelluloses were continuous, there was still a problem of collinearity among the spectral variables. To solve this problem, a CARS algorithm was adopted to select characteristic spectral bands from the ranges. The selected characteristic spectral bands are shown in Fig. 6.

Figure 6 shows that many of the selected characteristic bands of lignocelluloses overlap, especially for those of cellulose and hemicellulose at 1369, 1408, 1415, 1430, 1457, 1595, 1634, 1667, 1696, 1820, 1829, and 1883 cm^−1^. Specifically, the selected band 1369 cm^−1^ for cellulose is very close to the 1368 cm^−1^ selected for hemicellulose, which corresponds to a CH_3_ symmetrical angular vibration of cellulose and hemicellulose [43]. The overlapping bands of 1415 and 1412 cm^−1^ were assigned to the symmetrically stretching vibration of COOH. The selected band of 1430 cm^−1^ for cellulose and hemicellulose is closed related to the bending vibration of CH_2_ in olefin, and the selected bands of 1457 cm^−1^ correspond to a CH_3_ asymmetrical angular vibration in cellulose and a CH_2_-bending vibration in hemicellulose. The selected bands of 1529, 1609, 1610, 1633, and 1689 cm^−1^ for lignin overlapped with those of hemicellulose. In particular, the band at 1610 cm^−1^ was attributed to the stretching vibration of C=C plus the asymmetric-stretching vibration of COOH in the aromatic ring that is associated with cellulose, hemicellulose, and lignin. The above results indicate that the overlapping of lignocellulose characteristic bands is a phenomenon that is common with infrared band assignment [44].

The assignments of the other important feature bands to the structural constituents were as follows: 1373 cm^−1^ is associated with C–H deformation in cellulose and hemicelluloses [45], 1425 cm^−1^ represents CH_2_ scissor vibration in cellulose and hemicellulose [46], 1504 cm^−1^ is a diagnostic band for C=C-stretching vibration of the aromatic rings in lignin [47], 1508 and 1605 cm^−1^ are attributed to aromatic skeleton vibration in cellulose [8, 48], and 1735 cm^−1^ reflects the C=O-stretching vibration of carboxyl and acetyl groups in hemicellulose [49–51]. For the band of 1735 cm^−1^, the lignocellulosic component it contributes to establish regression model does not conform with its actual assignment. Because the priority of CARS algorithm is to select the optimal feature bands combination to establish the regression model with minimal root mean square error of cross validation, the selected characteristic band just has correlation with the lignocellulosic component, instead of reflects the functional group of the lignocellulosic component exactly. These band assignments are summarized in Table 5.

**Table 5:** FTIR-band assignments for the lignocellulosic components

Wavenumbers (cm^−1^)	Functional group	Polymer
1157	C–O–C asymmetrical-stretching vibration	Cellulose and hemicellulose
1369	CH_3_ symmetrical angular vibration	Cellulose and hemicellulose
1373	C–H deformation	Cellulose and hemicelluloses
1415	COOH groups symmetrically stretching vibration	Cellulose and hemicellulose
1425	CH_2_ scissor vibration	Cellulose and hemicellulose
1430	CH_2_ bending vibration in olefin	Cellulose and hemicellulose
1457	CH_3_ asymmetrical angular vibration CH_2_ bending vibration	Cellulose and hemicellulose
1504	C=C-stretching vibration in aromatic rings	Lignin
1508, 1605	Aromatic skeleton vibration	Cellulose
1610	C=C-stretching vibration COOH groups stretching vibration in aromatic ring	Cellulose, hemicellulose and lignin
1735	C=O-stretching vibration in ester groups	Hemicellulose

**Fig. 7:**
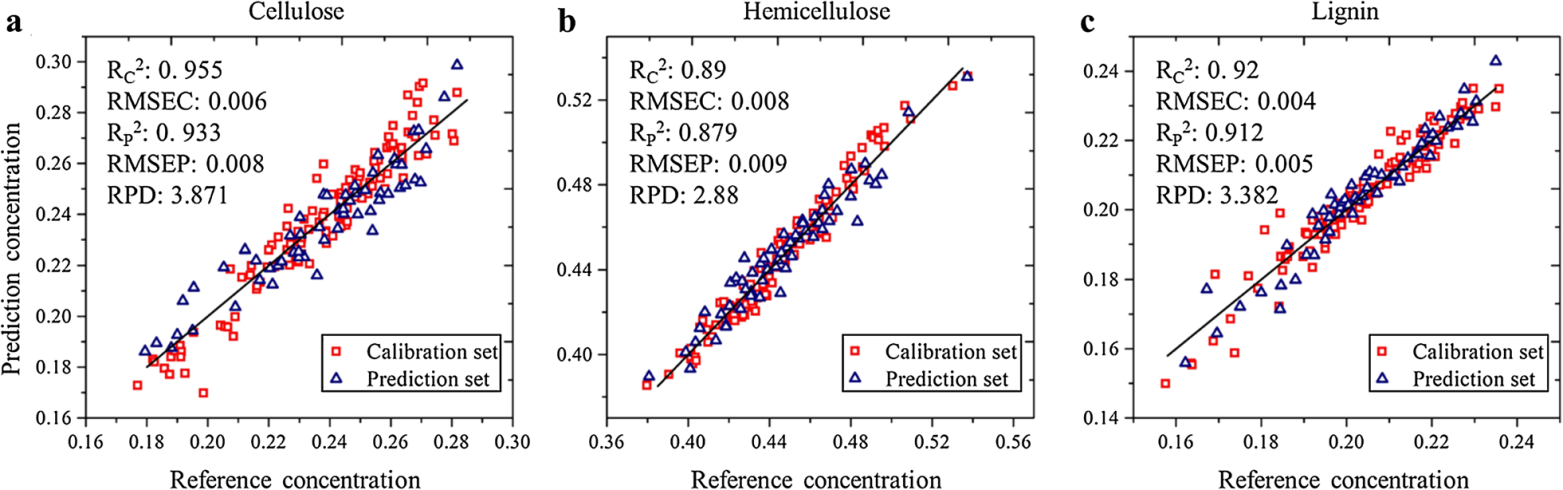
Scatter plots of the reference concentrations versus the prediction concentrations. **a** Cellulose, **b** hemicellulose, **c** lignin. Samples of different sets were marked with different symbols: the square and triangle denote the calibration set and prediction set, respectively

After selection of characteristic spectral bands, a series of PLSR models were developed based on these bands, and the related results are shown in Fig. 7. Comparing Fig. 7 with Table 4 demonstrates that an obvious improvement was achieved by models based on the selected bands, and the dimension of independent variables (spectral bands) has been reduced to less than 5% of the full-range spectroscopy. It can be concluded that these selected spectral bands represented fingerprints characteristic of lignocellulose determination.

### Spectral transfer and chemical imaging analysis of microstructure

#### Spectral transfer

**Fig. 8:**
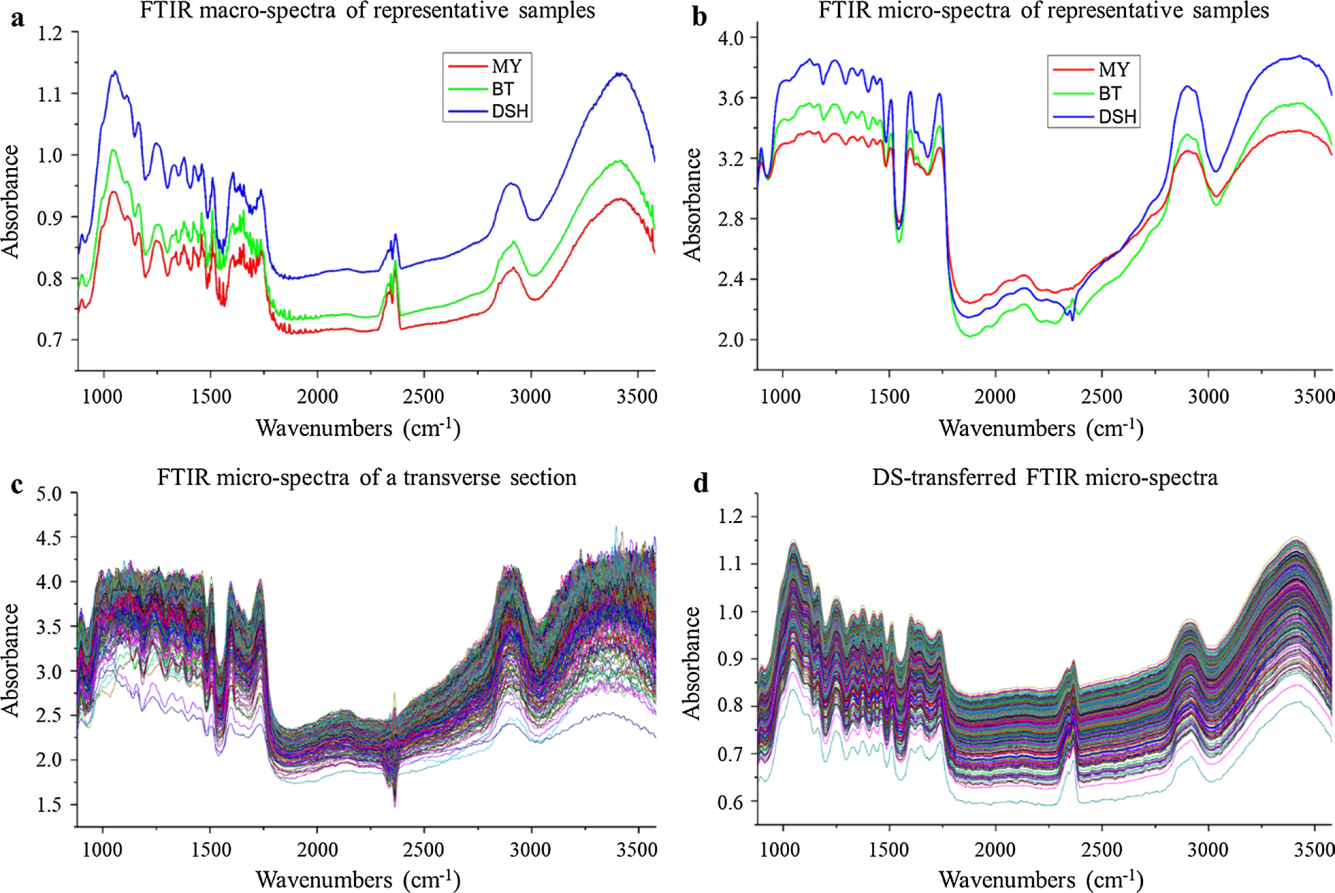
Corresponding spectra for DS transfer. **a** Representative of macro-spectra, **b** representative of micro-spectra, **c** original micro-spectra, **d** transferred micro-spectra based on DS algorithm

The representative spectra derived from master and slave instruments are illustrated in Fig. 8a, b, respectively. Although similar absorption peaks are observed in the overall spectral curves from the two instruments, differences of spectral responses in some regions can be easily observed. These differences may result from factors such as variations in the sample surface texture, granularity, spectral resolution, lateral resolution, and the area of illumination. For example, the samples for the FTIR macro-spectra measurement are powders with size between 250 and 380 μm [24–26], whereas the FTIR micro-spectra were acquired from transverse sections of tissues with vascular bundle and parenchyma cell with spatial resolution of 10 μm. To overcome the barriers between different intruments, the representative spectra (Fig. 8a, b) obtained according to the method in section “Spectral transfer by direct standardization” were adopted to calculate the transfer matrix based on the DS algorithm, and then, the original FTIR micro-spectra acquired by slave instrument shown in Fig. 8c could be transferred via multiplication with the transfer matrix. Compared with the original micro-spectra, the signal-to-noise ratio of the transferred FTIR micro-spectra (Fig. 8d) is obviously improved, which indicates that model transfer can improve the low SNR caused by high lateral resolution in FTIR micro-spectral measurement [35].

#### Chemical imaging analysis of microstructure

After model transfer, the developed PLSR models were applied to the transferred FTIR micro-hypercubes in a pixelwise manner to generate the lignocellulose distribution maps of the bamboo transverse sections.

**Fig. 9:**
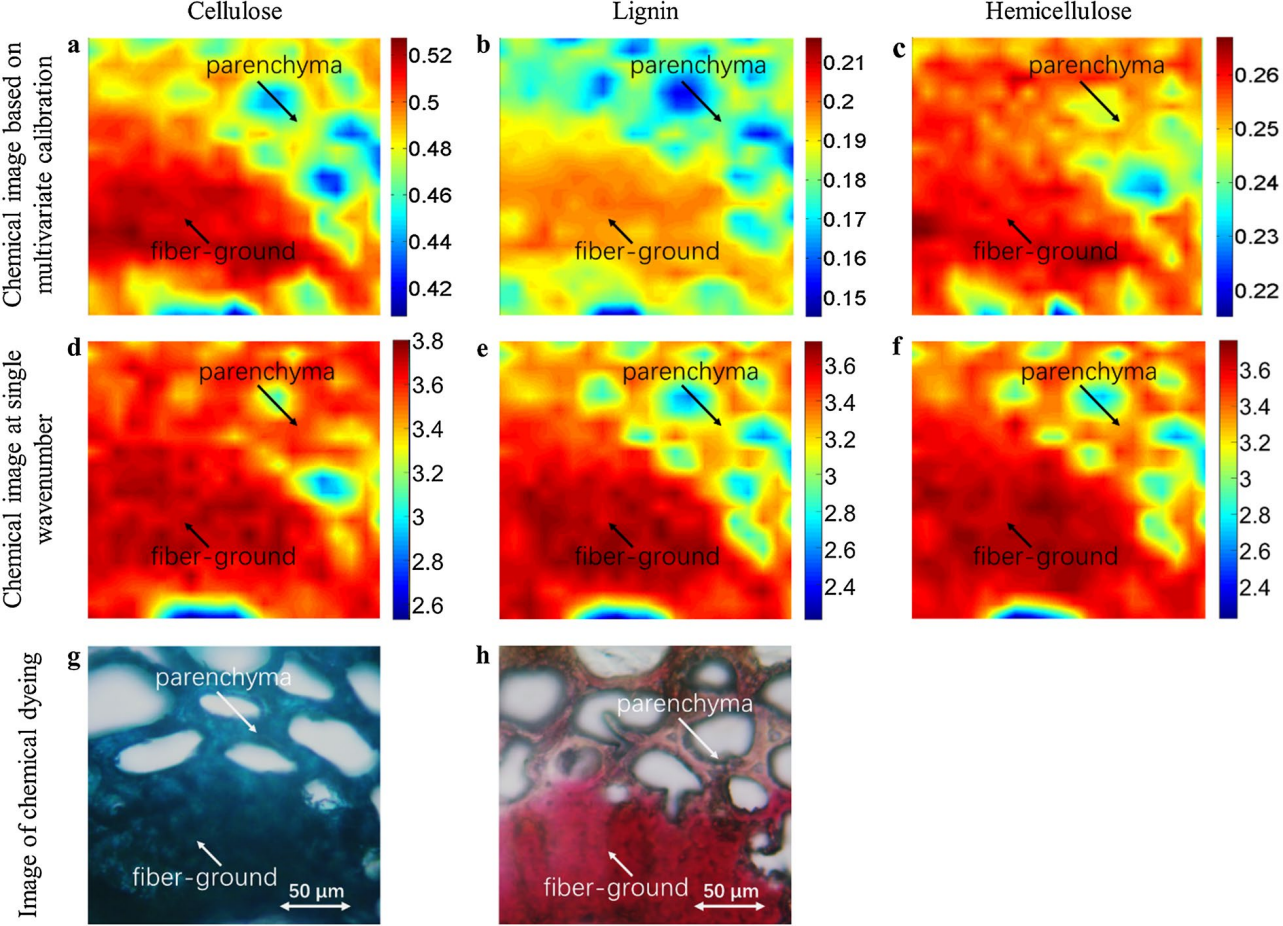
Results of the three chemical imaging methods. The first row shows the chemical images based on multivariate calibration model coupled with calibration transfer (**a**–**c**), and the color legend on the right side of the figure represents the concentration of lignocellulose; the second row shows the chemical images with single band (**d**–**f**) and the color legend represents the spectral intensity at well-defined characteristic band; and the third row shows the result of chemical dyeing method (**g**, **h**). The slice in **g** is dyed with methylene blue (AR, Sinopharm Chemical Reagent Corporation, China), and the slice in **h** is dyed with phloroglucinol (AR, Sinopharm Chemical Reagent Corporation, China). The first column shows the distribution of cellulose (**a**, **d**, **g**); the second column shows the distribution of lignin (**b**, **e**, **h**); and the third column shows the distribution of hemicellulose (**c**, **f**). For the pseudo-color maps in the first row, the numbers on the right side of the color bar indicate the percentages of lignocellulosic content; for the pseudo-color maps in the second row, the numbers on the right side of the color bar indicate the absorbance intensity

To evaluate the performance of the chemical imaging of lignocelluloses based on multivariate calibration analysis coupled with calibration transfer, chemical dyeing and single-intensity chemical imaging methods were applied, and the imaging results of the three methods are shown in Fig. 9. To achieve satisfactory dyeing effect, small cubes of moso bamboo were fixed, dehydrated, and embedded in paraffin, after which, sections with thickness of 2 μm were obtained by a rotary ultramicrotome (11800 Pyramitome, LKB Bromma, Sweden). For chemical images at single bands, 1157 cm^−1^ for the C–O–C vibration in cellulose and hemicellulose, 1504 cm^−1^ for aromatic skeleton in lignin [52], and 1734 cm^−1^ for unconjugated C=O in hemicelluloses were selected to map the lignocellulose distributions in bamboo transverse sections, as shown in Fig. 9d–f. Comparing the imaging results for cellulose in the first column, the distributions of cellulose in Fig. 9a, g are very similar, whereas the distribution region and content of cellulose in Fig. 9a are obviously smaller than shown in Fig. 9d, which shows the total distribution of cellulose and hemicellulose with chemical image at 1157 cm^−1^. It can be concluded that the chemical imaging method for cellulose based on multivariate calibration is more effective, and it can also avoid the interference of overlapping functional groups in the single-band imaging. It is worth noting that the chemical images of lignocelluloses (Fig. 9a, b) based on multivariate calibration analysis can provide quantitative concentrations of lignocelluloses in each pixel, as shown in the color legend on the right side of the figure, rather than the semi-quantitative analysis by chemical dyeing and single-intensity chemical imaging methods. In terms of lignin in the second column, the distributions of lignin in Fig. 9b, h maps are very similar, and the distribution region and content of lignin in Fig. 9b are obviously smaller than shown in Fig. 9e at 1504 cm^−1^, which is assigned to the aromatic skeleton to map lignin. This may be because the chemical image based on the selection of 1504 cm^−1^ for the aromatic skeleton contains not only lignin, but also other aromatic substances in bamboo. It can be concluded that the chemical imaging method of lignin based on multivariate calibration is more effective. Finally, the chemical imaging of hemicellulose, Fig. 9c, is compared with Fig. 9f, which is the spectral image for the peak at 1734 cm^−1^. This peak was selected for the ester group-stretching vibration in hemicelluloses [53–55]. It is worth noting that this band of 1734 cm^−1^ is also detected in dioxane lignin of spruce (G) and eucalyptus (G, S) [56]. It is observed that the content of lignin shown in Fig. 9f is obviously higher than that for the visualization of lignin in Fig. 9c, especially in the fiber strand. However, the chemical image in Fig. 9f is very similar with the lignin distribution in Fig. 9e, which indicates that the chemical imaging at 1734 cm^−1^ displays the total distribution of hemicellulose and lignin. To ascertain the difference of imaging performance based on the different methods, analysis of variance for the significance of the regression model versus a single band was performed. When conducting the analysis, a factor with two levels was set up with nine replicates at each level. Specifically, the two levels corresponded to the types of chemical imaging data, namely, the fitting values of the regression model and the absorbance intensity of single band, and the nine replicates refer to 3 sites 3× components. The obtained *p* value of 0 demonstrates that the imaging performance via regression model is completely different from that of the single band.

It can be concluded that it is difficult to obtain ideal chemical distributions of the components of interest using single-intensity chemical imaging methods, because the well-defined bands overlap those of other compounds. In contrast, the chemical imaging method based on the multivariate calibration model coupled with calibration transfer could provide a more powerful mean of detecting the chemical micro-distribution of the components of interest.

It is worth noting that there is an obvious distribution difference between cellulose and hemicellulose in the chemical imaging of bamboo transverse section based on multivariate calibration model coupled with model transfer (Fig. 9a, c). This difference is not shown in the single-band chemical imaging (Fig. 9d, f) of cellulose and hemicellulose, because their characteristic FTIR peaks often overlap, which results in very similar chemical images. It can be concluded that the chemical imaging strategy proposed in the present study could effectively reveal the difference in the distributions of cellulose and hemicellulose by mapping a set of the characteristic peaks of cellulose and hemicellulose that were derived from the multivariate calibration model. Moreover, this chemical imaging strategy can effectively reduce biases due to interferences in the single-band patterns compared to the single-intensity maps.

**Fig. 10:**
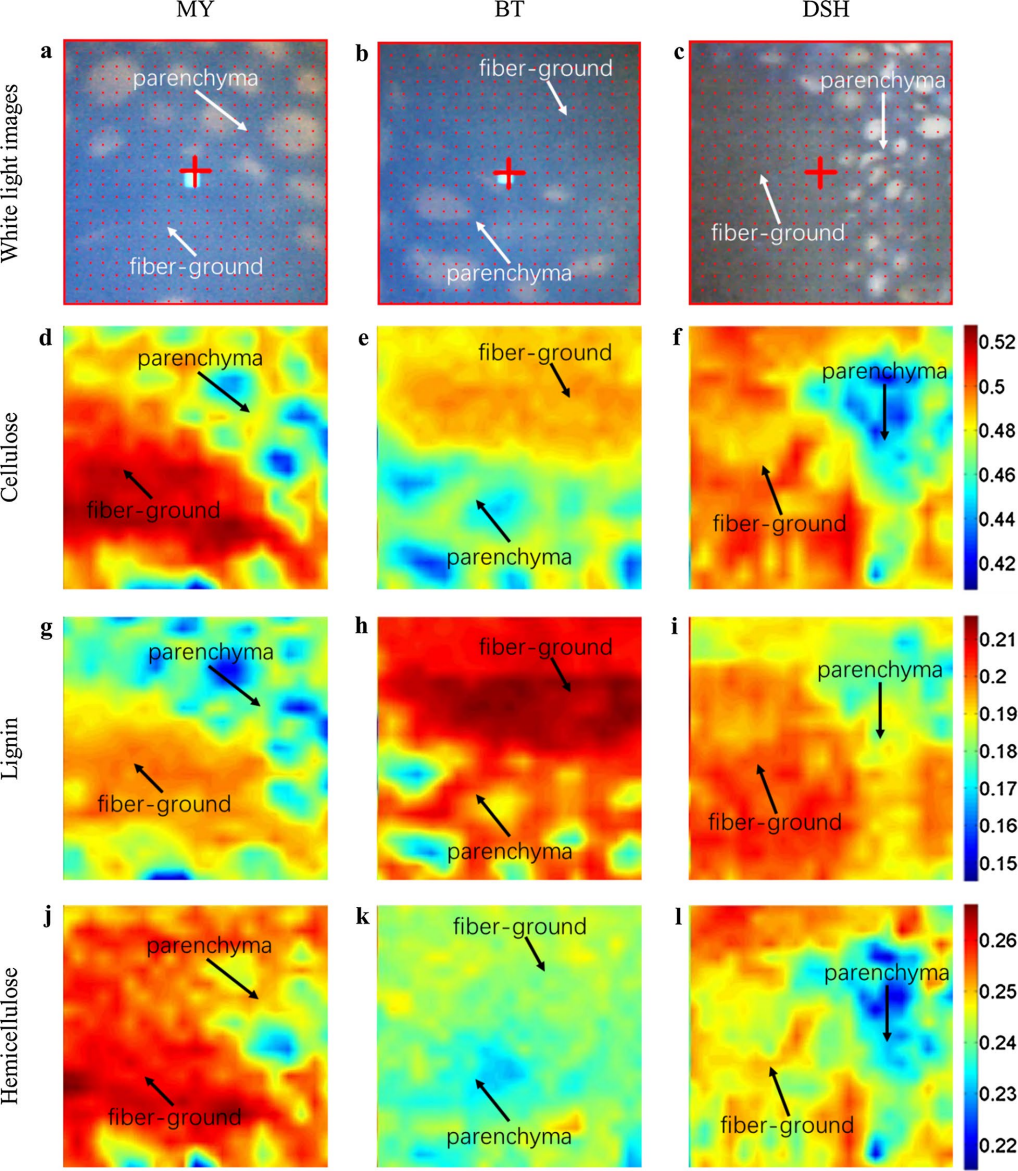
Results of the chemical imaging method based on multivariate calibration model coupled with calibration transfer. White light images of three transverse sections corresponding to MY, BT, and DSH (**a**–**c**, respectively); cellulose distribution images corresponding to MY, BT, and DSH (**d**–**f**, respectively); lignin distribution images corresponding to MY, BT, and DSH (**g**–**i**, respectively); and hemicellulose distribution images corresponding to MY, BT, and DSH (**j**–**l**, respectively). The numbers on the right of the color bar indicate the percentages of the lignocellulosic content

The white light images of three transverse sections show the anatomical structures of the bamboo transverse sections, including the fiber strand, parenchymal cells, and their boundary regions. Figure 10d–f shows that cellulose is mainly concentrated in the fiber strand and appears to decline in the transition region from the fiber strand to parenchymal cells. In the fiber strand, most of the fibers are thick-walled fibers with small cell cavities. The thick cell wall of fiber strand is mainly composed of cellulose, whereas the periphery of parenchymal cells consists of uniformly distributed cellulose. The spatial distribution of lignin is shown in Fig. 10g–i, which shows that lignin is mainly concentrated in the fiber strand, with less throughout the parenchymal cells. This is to be expected, because the fiber strand is the main support structure of bamboo, and high lignification can improve the mechanical strength of bamboo. Figure 10j–l shows the spatial distribution of hemicellulose and shows that in contrast to cellulose and lignin, hemicellulose is distributed relatively uniformly throughout the tissue. In general, the distributions of lignin and cellulose are higher than that of hemicellulose in the fiber strand, whereas the relative content of hemicellulose is higher in the parenchyma cells than in the fiber strand.

## Conclusion

This study proposed a new method for quantitative FTIR micro-chemical imaging of lignocelluloses. The method combines quantitative multivariate calibration analysis with model transfer based on the FTIR micro-spectroscopic technique. By correcting the difference between the FTIR macro- and micro-spectroscopic data using the DS transfer algorithm, a quantitative lignocellulosic model that was developed from the FTIR macro-spectroscopy could be introduced to the corrected FTIR micro-spectroscopic hypercube in a pixelwise manner to generate a quantitative microspatial distribution of the lignocellulosic constituents of bamboo. This approach could achieve a quantitative visualization of the microstructure of a specific chemical index, rather than semi-quantitative analysis based on mapping of single band. Furthermore, this approach could avoid a catastrophic problem that results from the spectral overlap between different absorption bands and, to a certain extent, reduce the fatal interference of random errors such as band shift, baseline correction error, and spectral artifacts in single-band chemical imaging.

FTIR micro-spectroscopic imaging coupled with multivariate calibration analysis has the ability to reveal the quantitative distribution of lignocellulosic compounds in the tissue structure of transverse sections of bamboo. The chemical images show that the thick cell wall of the fiber strand is mainly composed of cellulose, followed by lignin, which differs from the components of the parenchyma cells. In contrast to cellulose and lignin, hemicellulose is distributed relatively uniformly throughout the tissue. The integration of FTIR macro- and micro-spectroscopic imaging techniques can provide comprehensive information for the use of the moso bamboo resource, such as developing biofuels and biosynthetic materials.

This is the first publication to bridge the macro quantitative homogeneity determination with the micro-tissue compositional heterogeneity associated with the anatomical structures through an effective integration of FTIR macro- and micro-spectroscopic imaging techniques. With the merits of quantitative determination and micro-distribution visualization, this new chemical imaging technique should be helpful in the optimization of the use of other types of biomass fuel and materials, such as lipid accumulation in microalgae [57].

## Abbreviations

AR: analytical reagent
BT: Baitanao
CARS: competitive adaptive reweighted sampling
DS: direct standardization
DSH: Daishi
FTIR: Fourier transform infrared
GR: guaranteed reagent
iPLS: interval partial least squares
IR: infrared
MCS: Monte Carlo sampling
MRT: multiple range test
MY: Maoyang
NIR: near infrared
PLSR: partial least-squares regression
*R*_C_^2^: coefficients of determination from the calibration sets
*R*_P_^2^: coefficients of determination from the prediction sets
RMSEC: root mean square error of calibration
RMSEP: root mean square error of prediction
RMSEV: root mean square error of cross validation
RPD: ratio of standard deviation of prediction set to standard deviation of prediction error
SP: spectral purity
STD: standard deviation
